# Neurological Complications in Post-COVID-19 Infected Patients: A Case Series

**DOI:** 10.7759/cureus.32374

**Published:** 2022-12-10

**Authors:** Kartheek Minna, Nikhil Doshi, Bhumika Vaishnav, Aniruddh N Wadivkar, Farhanulla Basha

**Affiliations:** 1 General Medicine, Dr. D. Y. Patil Medical College, Hospital and Research Centre, Pune, IND

**Keywords:** acute transverse myelitis (atm), guillain-barre syndrome, neuropathy, novel coronavirus, covid-19

## Abstract

In December 2019, the novel coronavirus severe acute respiratory syndrome coronavirus 2 (SARS-CoV-2), which is the causative agent of coronavirus disease 2019 (COVID-19), emerged in China and quickly spread to other countries. COVID-19 infection can present in a variety of ways, ranging from mild upper respiratory illness with no symptoms to severe acute respiratory distress syndrome with multiorgan involvement and death. With increasing frequency, new associations and clinical complications, such as thrombotic states, mucormycosis, and others have been reported. Neurological complications can occur during infection, during the immediate recovery period, or as late sequel of infection in COVID-19. We present an intriguing case series of neurological complications following COVID-19 pneumonia.

## Introduction

A novel coronavirus disease 2019 (COVID-19) was detected in Wuhan City, Hubei Province, China, on 31 December 2019 [1]. At the onset of the disease, the most common symptoms are fever, cough, dyspnea, myalgia, headache, and diarrhea. Although severe acute respiratory syndrome coronavirus 2 (SARS-CoV-2) is a respiratory virus, several reports have been published regarding COVID-19 disease spectrum involvement in the central and peripheral nervous systems [2,3].

Meningitis, encephalitis, myelitis, central nervous system (CNS) vasculitis, acute disseminated encephalomyelitis, Guillain-Barre syndrome (GBS), and other acute neuropathies are frequently observed neurological complications [2,4].

GBS is a relatively uncommon complication of COVID-19. GBS was identified as a complication in many SARS-CoV-2 cases in an Italian study that observed about 1,200 patients over a period of one month [5]. In this case series, we describe two cases of GBS and one case of acute transverse myelitis in patients who were infected with or had a recent history of COVID-19 infection.

## Case presentation

Case 1

A 48-year-old female presented with acute onset, progressive weakness of limbs (lower limb>upper limb) for six days. She was diagnosed with COVID-19 infection 20 days ago. The COVID reverse transcriptase-polymerase chain reaction (RT-PCR) was negative on admission. The neurological examination revealed motor weakness in both upper and lower extremities (Medical Research Council {MRC} score: 2/5 proximally, 3/5 distally for upper extremities, and 1/5 proximally, 2/5 distally for lower extremities) and absence of deep tendon reflexes. Additionally, facial diplegia was observed.

Table 1 shows the laboratory investigations, the WBC count was elevated, as well as the erythrocyte sedimentation rate, and the c-reactive protein level. Liver and renal function tests were normal. Magnetic resonance imaging (MRI) of the cervical spine and brain was normal. The computed tomography (CT) of chest revealed diffuse consolidations and ground-glass opacities in both lungs, with a CT severity index of 15/25 and a coronavirus disease 2019 reporting and data system (CORADS) of 4. The nerve conduction velocity study revealed a predominance of motor, axonal, and demyelinating polyradiculoneuropathy in all four limbs.

**Table 1:** Laboratory investigations of case 1.

Laboratory parameters	Laboratory values	Reference range
Hemoglobin	13.6 g/dL	11.6-15.0 g/dL
White blood count	13,200 cells/cumm	4,000-10,000 cells/cumm
Erythrocyte sedimentation rate	67 mm/h in the first hour	Female under 50 years: up to 20 mm/h. Female 50-85 years: up to 30 mm/h. Female >85 years: up to 42 mm/h.
C-reactive protein	46 mg/dL	<2.0 mg/dL
Total bilirubin	0.32 mg/dL	0.22-1.20 mg/dL
Serum glutamic oxaloacetic transaminase	24 U/L	8-48 U/L
Serum glutamic pyruvic transaminase	11 U/L	7-55 U/L
Alkaline phosphatase	77 U/L	40-129 U/L
Urea	39 mg/dL	17-49 mg/dL
Creatinine	0.76 mg/dL	0.6-1.35 mg/dL

Table 2 shows cerebrospinal fluid analysis which shows albuminocytological dissociation (Brighton criteria level 1). A definitive diagnosis of acute inflammatory demyelinating polyradiculoneuropathy (acute motor axonal neuropathy {AMAN} variant) post-COVID-19 pneumonia was made. The patient received 0.40 g/kg/day of intravenous immunoglobulin and supportive care for five days. On day two of admission, she developed respiratory paralysis and was placed on a mechanical ventilator. Over the next two weeks, the patient's general condition, motor weakness, and respiratory paralysis gradually improved. On day 12, she was extubated, and on day 30, she was discharged from the hospital.

**Table 2:** Cerebrospinal fluid analysis of case 1.

Laboratory parameters	Laboratory values	Reference range
Total leukocyte count (TLC)	2 cells/cumm	0-500 cells/cumm
Protein	209 mg/dL	15-45 mg/dL
Glucose	56 mg/dL	>60 mg/dL
Adenosine deaminase (ADA)	1.74 U/L	0-39 U/L

Case 2

A 22-year-old male patient came with complaints of weakness in both lower limbs, associated with pain and tingling sensation for five days. Two weeks ago, he had a fever and tested COVID RT-PCR positive. His COVID RT-PCR was negative on admission to our hospital. Weakness in the limbs began nine days after the resolution of upper respiratory symptoms. On neurological examination, the patient had MRC score of 3/5 in the upper limbs and 1/5 in the lower limbs with absent deep tendon reflexes. Table 3 shows the laboratory investigations of the patient. His hemogram, liver, and renal function tests were normal, c-reactive protein level was elevated. MRI brain and spine were normal.

**Table 3:** Laboratory investigations of case 2.

Laboratory parameters	Laboratory values	Reference range
Hemoglobin	14.6 g/dL	13.2-16.6 g/dL
White blood count	7,800 cells/cumm	4,000-10,000 cells/cumm
C-reactive protein	207.47 mg/dL	<2.0 mg/dL
Total bilirubin	0.41 mg/dL	0.22-1.20 mg/dL
Serum glutamic-oxaloacetic transaminase	35 U/L	8-48 U/L
Serum glutamic pyruvic transaminase	32 U/L	7-55 U/L
Alkaline phosphatase	65 U/L	40-129 U/L
Urea	36 mg/dL	17-49 mg/dL
Creatinine	0.82 mg/dL	0.6-1.35 mg/dL

Table 4 shows CSF analysis depicting albuminocytological dissociation. The nerve conduction study revealed demyelinating motor and sensory polyradiculoneuropathy (Brighton criteria level 1). The final diagnosis was acute inflammatory demyelinating polyradiculoneuropathy (AIDP) post-COVID-19 infection. The patient was started on intravenous immunoglobulin (IVIG - in the dose of 0.4 g/kg/day) for five days. On day two of admission, the patient was put on an invasive mechanical ventilator for hypoxia due to respiratory muscle paralysis. Over the next 10 days, the patient's general condition gradually improved. On day 20 of his admission, he was discharged with an MRC score of 4/5 in all four limbs.

**Table 4:** Cerebrospinal fluid analysis of case 2.

Laboratory parameters	Laboratory values	Reference range
Total leukocyte count (TLC)	6 cells/cumm	0-500 cells/cumm
Protein	230 mg/dL	15-45 mg/dL
Glucose	52 mg/dL	>60 mg/dL
Adenosine deaminase (ADA)	1.6 U/L	0-39 U/L

Case 3

A 40-year-old male presented to the emergency department with progressive bilateral lower limb weakness, urinary retention, and constipation for three days. The patient reported suffering from mild COVID-19 infection 10 days prior to this (home quarantined). He tested positive for COVID RT-PCR. There was no history of fever, rash, oral or genital ulcers, joint pain, photosensitivity, and high-risk behavior. On examination, he had hypertonia, exaggerated deep tendon reflexes, motor weakness (power grade 3/5) in both lower limbs, and loss of proprioception with paraesthesia till umbilicus (T10 dermatome). Meningeal signs were absent. Table 5 shows the laboratory investigations. C-reactive protein level and erythrocyte sedimentation rate were both elevated. Serology for HIV, hepatitis B, and C was negative.

**Table 5:** Laboratory investigations of case 3.

Laboratory parameters	Laboratory values	Reference range
Hemoglobin	13.8 g/dL	13.2-16.6 g/dL
White blood count	6,900 cells/cumm	4,000-10,000 cells/cumm
C-reactive protein	41 mg/dL	<2.0 mg/dL
Erythrocyte sedimentation rate	67 mm/h in the first hour	Female under 50 years: up to 20 mm/h. Female 50-85 years: up to 30 mm/h. Female >85 years: up to 42 mm/h.
Total bilirubin	0.47 mg/dL	0.22-1.20 mg/dL
Serum glutamic-oxaloacetic transaminase	44 U/L	8-48 U/L
Serum glutamic pyruvic transaminase	43 U/L	7-55 U/L
Alkaline phosphatase	73 U/L	40-129 U/L
Urea	44 mg/dL	17-49 mg/dL
Creatinine	0.68 mg/dL	0.6-1.35 mg/dL

**Figure 1:**
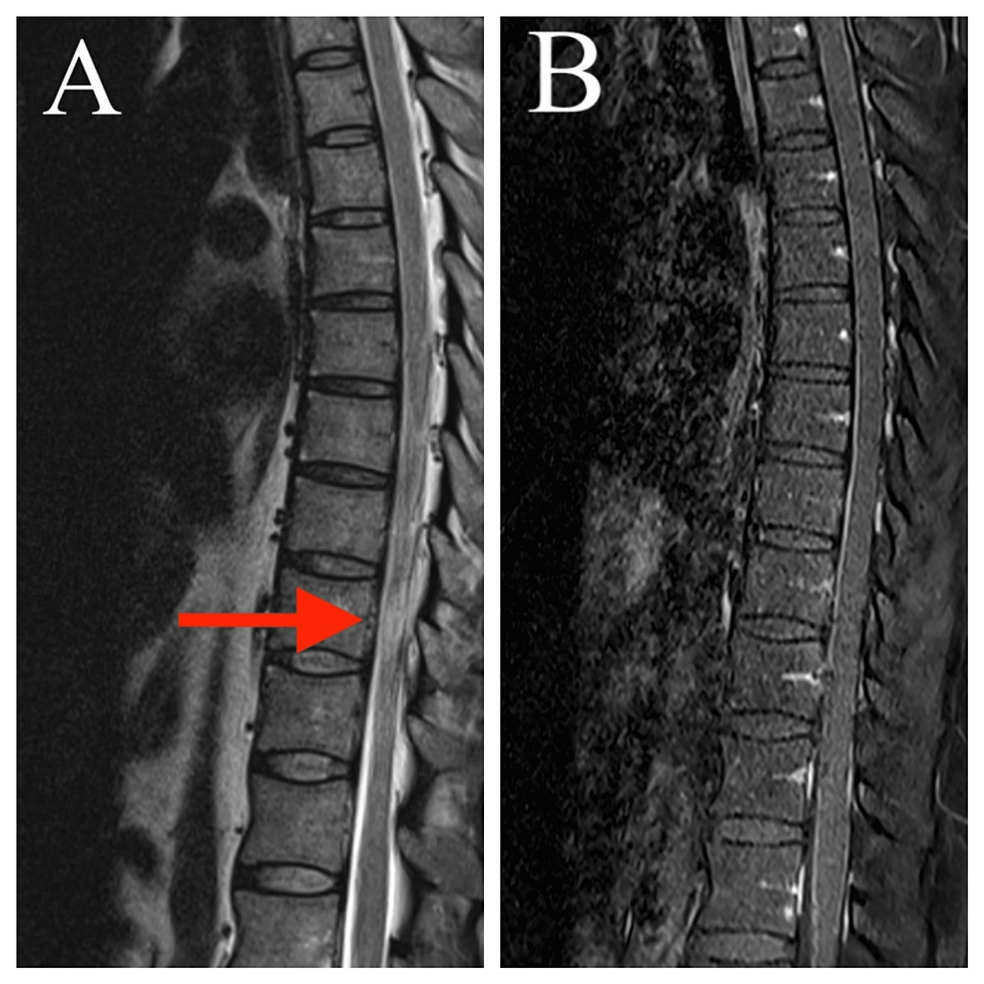
MRI thoracic spine sagittal (A) T2 shows hyperintense signal at T7-T10 cord level and (B) T1 contrast shows no post-contrast enhancement.

Figures 1a, 1b depict MRI of the thoracic spine sagittal T2 showing hyperintense signal in the long segment extending from T7 to T10 cord level without significant post-contrast enhancement suggestive of acute transverse myelitis. CSF analysis was normal and negative for oligoclonal bands. There was no evidence of optic neuritis on eye examination. Anti-NMO-MOG and anti-AQP4 were negative. Thus, neuromyelitis optica (NMO) was ruled out. ANA by IF was negative. CSF COVID-19 RT-PCR was negative. MRI brain plain was normal ruling out white matter autoimmune disorders. The final diagnosis was acute transverse myelitis (ATM) post-COVID-19 infection. He was treated with intravenous methylprednisolone at a dose of 1 g per day for five days. He improved clinically and regained power in lower limbs (grade 4/5) with intensive physiotherapy, and he was discharged on day 15 of admission.

In all three cases, the history of the patients was not suggestive of any other antecedent infections except SARS- CoV-2 infection. Also, they did not have any clinical features or history of any other organ involvement or infection.

## Discussion

The SARS-CoV-2 virus primarily targets the respiratory system via a fusion with the angiotensin-converting enzyme 2 (ACE2) receptor, however, neurological involvement is not uncommon and can complicate the clinical course [6].

GBS is a rapidly progressive, immune-mediated polyneuropathy that is frequently preceded by infection. Numerous infectious agents, including *Campylobacter jejuni*, Cytomegalovirus, Epstein-Barr, and Zika virus, have been linked to GBS [7]. The two patients in our case series had classic post-infectious, demyelinating GBS phenotypes.

Zhao et al. reported the first case of GBS following COVID-19 infection in a 61-year-old female who developed demyelinating polyneuropathy following a trip to Wuhan, China [8]. Additionally, Filosto et al. reviewed 24 cases of Guillain-Barré syndrome that could have been caused by COVID-19 [9].

Both patients in our case series developed GBS after recovering from mild COVID-19 disease. The unique characteristic of these patients was that both had mild COVID-19 infection without hypoxia or respiratory complications. Thus, it appears as though the pathogenesis of neurological involvement is distinct from that of respiratory involvement. The mechanism of association of GBS with COVID-19 remains unknown. GBS is an immune-mediated disease, and molecular mimicry may play a role [10]. Numerous mechanisms have been proposed, including direct spread through the cribriform plate, upregulation of the angiotensin-converting enzyme (ACE2) receptor on glial tissues, S-spike viral protein-mediated damage, and altered exosomal transport of viral particles to glial tissues [11].

However, the inflammatory cytokine surge that occurs in response to COVID-19 infection as a result of CD4+T cell activation is one of the most promising mechanisms for explaining indirect neuronal pathway damage that manifests as gradually increasing and then resolving weakness [12].

The mechanism of acute transverse myelitis post-COVID-19 infection is also unknown. The proposed mechanism for ATM is that SARS-CoV-2 may cause neuronal injury via hypoxic and immune-mediated mechanisms. Viral replication and increased ACE2 receptor activation in the CNS may initiate a systemic inflammatory response, resulting in increased blood-brain barrier permeability and immune-mediated CNS inflammation. This response is thought to be mediated by IL-6, a proinflammatory cytokine.

COVID-19 had a significant global impact, infecting millions of people and causing deaths. It has the potential to cause both acute and chronic neurological complications in a large number of patients. It is critical to continue investigating and comprehending the clinical manifestations and mechanisms of COVID-19. This will aid in the investigation of the pathogenesis of this novel disease and may result in the development of novel treatment strategies.

Additionally, follow-up of people infected with SARS-CoV-2 should include a thorough examination of the nervous system to rule out the development of late complications. A physician’s role is vital in the management of SARS-CoV-2 infection and its sequelae. This case is an attempt to educate the medical fraternity about the neurological complications and repercussions of the dreaded coronavirus.

## Conclusions

All physicians treating patients with COVID-19 should be aware of the possibility of post-COVID-19 neurological sequelae and should closely monitor these patients. Recognizing potential neurological complications in patients early on aids in prompt treatment and reduces morbidity associated with such complications.
